# Impact of maternal education, employment and family size on nutritional status of children

**DOI:** 10.12669/pjms.336.13689

**Published:** 2017

**Authors:** Aisha Iftikhar, Attia Bari, Iqbal Bano, Qaisar Masood

**Affiliations:** 1 Dr. Aisha Iftikhar, FCPS. Department of Peadiatrics Medicine, Children’s Hospital and Institute of Child Health, Lahore, Pakistan; 2 Dr. Attia Bari, DCH, MCPS, FCPS. Department of Peadiatrics Medicine, Children’s Hospital and Institute of Child Health, Lahore, Pakistan; 3 Dr. Iqbal Bano, FCPS. Department of Peadiatrics Medicine, Children’s Hospital and Institute of Child Health, Lahore, Pakistan; 4 Dr. Qaisar Masood, DCH, MCPS. Department of Peadiatrics Medicine, Nawaz Sharif Hospital, Lahore, Pakistan

**Keywords:** Family size, Malnutrition, Maternal education, Maternal employment

## Abstract

**Objective:**

To determine the impact of maternal education, employment, and family size on nutritional status of children.

**Methods:**

It was case control study conducted at OPD of children Hospital Lahore, from September 2015 to April 2017. Total 340 children (170 cases and 170 controls) with age range of six months to five years along with their mothers were included. Anthropometric measurements were plotted against WHO growth Charts. 170 wasted (<-2 SD) were matched with 170 controls (≥ -2 SD). Maternal education, employment and family size were compared between the cases and control. Confounding variables noted and dichotomized. Univariate analysis was carried out for factors under consideration i.e.; Maternal Education, employment and family size to study the association of each factor. Logistic regression analysis was applied to study the independent association.

**Results:**

Maternal education had significant association with growth parameters; OR of 1.32 with confidence interval of (CI= 1.1 to 1.623). Employment status of mothers had OR of 1.132 with insignificant confidence interval of (CI=0.725 to 1.768). Family size had OR of one with insignificant confidence interval (CI=0.8 -1.21). Association remained same after applying bivariate logistic regression analysis.

**Conclusion:**

Maternal education has definite and significant effect on nutritional status of children. This is the key factor to be addressed for prevention or improvement of childhood malnutrition. For this it is imperative to launch sustainable programs at national and regional level to uplift women educational status to combat this ever increasing burden of malnutrition.

## INTRODUCTION

Even at this day and age child malnutrition is one of the challenges being faced by developing countries.^1^,^2^ Pakistan is one of those countries, which are striving hard to overcome this ever-increasing burden of malnutrition for many decades.^3^

Child malnutrition is considered as the key risk factor for illness and death, contributing to more than half deaths of children globally.^4^ It not only increases the frequency and severity of infections but makes the children vulnerable to death from common infections.^5^ Under nutrition especially in first 1000 days of life lead to stunted growth and impaired cognitive ability and poor educational attainment.^6^,^7^

One in every nine people remained undernourished predominantly in developed countries in years 2014-2016.^8^ Over 50% children in South Asia are malnourished.^9^ Half of the world’s malnourished children reside in Pakistan, India, and Bangladesh. In South East Asia Pakistan has second highest infant and Childhood mortality.^10^ According to most recent data in Pakistan 43.7% of children were stunted, 15.1% were wasted and 31.5% were underweight. Conditions being worse in rural than in urban areas.^11^ Severe stunting was present in 21.9% of cases while 5.8% had severe wasting and 11.6% were underweight.^11^

Effect of maternal education on nutritional status of children has been demonstrated decades ago and is far reaching than any other determinant.^12^,^13^ Ability to acquire the health knowledge, following the recommended feeding practices and increased command over resources are the suggested conduits through which maternal education influences the child health.^14^

In addition to the illiteracy, educated mothers having high educational achievements also have children who are undernourished. This highlights the importance of time and attention devoted to children, which employed and highly educated woman cannot provide, depriving their children from the benefits of their education.^13^

A number of studies have been done to elucidate the demographic, socioeconomic, maternal and political factors, which are considered to be main determinants of malnutrition in children^15^, but at national level there is paucity of literature regarding the importance of maternal education and employment level. Through this study we want to highlight the importance of these two determinants and also want to see the effect of family size on nutritional status of children. We conducted this study with the objective to determine the impact of maternal education, employment, and family size on the nutritional status of children.

## METHODS

It was case control study conducted at OPD of The Children’s Hospital Lahore, from September 2015 to April 2017. Approval from Institutional Review Board was taken before commencement of study. Questionnaire Performa was used. After informed consent children of both genders with age range of six months to five years old, visiting outpatient department were included. Children having any chronic disease (e.g.; congenital heart disease, renal failure, chronic lung disease or chronic liver disease) were excluded from the study. Three hundred and forty children (170 cases and 170 controls) with their mothers were included in study. Anthropometric measurement of children was done and plotted against WHO growth Charts. Those children whose weight for height was falling from median to at or above -2 SD score of WHO growth chart were considered control. Those children whose weight for height was falling <-2 SD were considered as cases.

Their mothers were interviewed for determinants of under nutrition under consideration i.e.; education, employment and family size. Education level was classified as educated or uneducated. Uneducated was defined as those who have not attended the school or could not read and write their name. Educated mothers were defined as those who could read and write their name or have attended school. Confounding factors like father’s education level, maternal age, child’s gender, under five children, family size rural or urban residence, joint or nuclear family, separated or single parent were also noted. Data was analyzed by using statistical soft-ware SPSS 20. Determinants of malnutrition under consideration i.e. education and employment status of mothers and family size were compared between the cases and control. The potential confounding variables were dichotomized. Father’s education as educated or not, maternal age as < 30 years or >30 years, child’s gender, belonging to urban and rural area, family size was grouped as 5 or <5 and other group >5, under five children two or less and >2 children, parent’s status as living together or separated, family system as nuclear (husband, wife and children only) or joint (husband, wife, children, and maternal or paternal grandparents).

Univariate analysis was carried out for factors under consideration i.e.; maternal education, employment and family size to study the association of each factor. Odds ratio (OR) and 95% confidence interval was estimated for each of the factor. The simultaneous effects of maternal education, maternal employment and family size with other confounding factors like father’s education, maternal age, child’s gender, under 5 children, family size, rural or urban background, family system, parents status were analyzed using logistic regression analysis to study the independent association of these factors with the nutritional status. A p-value of less than 0.05 and an OR with a 95% confidence interval (CI) that did not include one was considered significant.

## RESULTS

In our study, we incorporated 340 children with age range of six months to five years with 170 cases and 170 controls. Mean age of children was 21.64 ± 15.4 months. The gender distribution was uniform with 52% of cases and controls male. Comparative anthropometric, socio-demographic characteristics of study population are shown in Table-I.

**Table-I:** Comparative anthropometric, socio-demographic characteristics of study population.

*Anthropometry & Demographics*	*Case*	*Control*
Weight (mean & sd)	6.38 ± 2.23	10.43 ± 3.9
Height (mean & sd)	71.56± 10.67	79.21± 14.21
***Mother’s Education***		
Educated	88(51.8%)	111(65%)
Un-Educated	82(48.2%)	59(34.7%)
***Father’s education***		
Educated	121(71.2%)	124(72.9%)
Un-educated	49(28.8%)	46(27.1%)
***Employment status***		
Employed	9(5.3%)	7(4.1%)
Un-employed	161(94.7%)	163(95.9%)
***Family size***		
≤ 5	56 (32.9%)	59(34.7%)
> 5	114 (67.1%)	111(65.3%)
***Maternal age***		
< 30 yrs	108 (63.5%)	94(55.3%)
> 30 yrs	62(36.5%)	76(44.7%)
***Under 5 children***		
≤ 2	143(84.1%)	145 (85.3%)
> 2	27 (15.9%)	25 (14.7%)
***Residential Area***		
Rural	60(35.3%)	38(22.4%)
Urban	110(64.7%)	132(77.6%)
***Family system***		
Joint	106(62.4%)	96 (56.5%)
Nuclear	64(37.6%)	74 (43.5%)
***Parent-status***		
Separated	1(0.6%)	2(1.2%)
Living together	169(99.4%)	168(98.8%)
***Income***		
≤10,000 Pak-Rupees	113(66.5%)	85(50.0%)
>10,000 Pak-Rupees	57(33.5%)	85(50.0%)

Literacy of fathers surpassed the mother’s as out of 340 children 199 mothers (58%) were educated as compared to 245(72%) fathers who were educated. Comparison of maternal education, employment, and family size was done between cases and controls and odds ratio with confidence interval was determined. Abut 51.8% mothers were educated in cases as compared to 65.3% of educated mothers in control with OR of 1.316 and (CI= 1.1 to 1.623). Table-II Employment status of mothers was found to be very low with only 9% of employed mothers in cases as compared to 7% in control with OR of 1.132 and insignificant CI of (CI=0.725 to 1.768). Third variable under consideration showed that 67.1% of cases had family size of more than five compared to 65.3% in control with OR of one with insignificant CI of (CI=0.8 - 1.21). To study the combined effects of different variables on the risk of being undernourished children bivariate logistic regression model was applied which showed no significant change in association which is depicted in Table-III.

**Table-II:** Crude odds ratio and confidence interval between under nutrition and risk factors.

*Category*			*Case*	*Control*	*OR (CI)*	*p-value*
Maternal education	Educated	199 (58%)	88(51.8%)	111(65%)	1.32 (1.1 – 1.62)	0.008
	Un-educated	141(42%)	82(48.2%)	59(34.7%)		
Father- education	Educated	245(72%)	121(71.2%)	124(72.9%)	1.045 (0.82–1.32)	0.405
	Un-educated	95(28%)	49(28.8%)	46(27.1%)		
Employment	Employed	16 (4.7%)	9(5.3%)	7(4.1%)	1.132 (0.725-1.768)	0.39
	Unemployed	324 (95.3%)	161(94.7%)	163(95.9%)		
Family size	≤ 5	115(34%)	56 (32.9%)	59(34.7%)	0.961 (0.765-1.207)	0.409
	>5	225(66.2%)	114 (67.1%)	111(65.3%)		
Maternal age	< 30 yrs	202(59.4%)	108 (63.5%)	94(55.3%)	1.2 (1-1.4)	0.075
	> 30 yrs	138 (40.6%)	62(36.5%)	76(44.7%)		
Under 5 children	≤ 2	288(84.7%)	143(84.1%)	145 (85.3%)	0.956 (0.718-1.273)	0.4
	>2	52 (15.3%)	27 (15.9%)	25 (14.7%)		
Residential Area	Rural	98 (28.8%)	60(35.3%)	38(22.4%)	1.34 (1.1 – 1.661)	0.006
	Urban	242 (71.2%)	110(64.7%)	132(77.6%)		
Family- system	Joint	202 (59.4%)	106 (62.4%)	96 (56.5%)	1.13 (0.91 – 1.41)	0.16
	Nuclear	138 (40.6%)	64(37.6%)	74 (43.5%)		
Parent status	Separated	3(0.9%)	1(0.6%)	2(1.2%)	0.6(0.14 – 3.11)	0.50
	Living together	337 (99.1%)	169 (99.4%)	168(98.8%)		
Income	≤10,000 Pak -Rupees	198 (58.2%)	113(66.5%)	85(50.0%)	1.4 (1.1-1.79)	0.001
	>10000 Pak- Rupees	142 (41.8%)	57(33.5%)	85(50.0%)		

**Table-III:** Estimates of simultaneous effect of various factors through logistic regression analysis.

*Factors*	*ODDS Ratio*	*95% CI*	*P- Value*
Maternal education	1.76	1.107-2.807	0.017
Employment	1.316	0.456 – 3.795	0.612
Family size	0.851	0.528 -1.371	0.508
Maternal age	1.603	0.981-2.621	0.06
Income	2.064	1.312 – 3.245	0.002
Father education	0.893	0.538 – 1.483	0.662
Parents status	0.279	0.023-3.412	0.318
Under 5 year	0.654	0.333-1.283	0.217

## DISCUSSION

Our study result established that low literacy rate of mothers was significantly associated with poor nutritional status of children. These results were consistent with results of studies conducted around the globe, e.g.; a case control study done in Africa shows strong impact of maternal literacy on child’s nutritional status.^16^ Another case control study proved that parental illiteracy was significantly associated with the risk to develop malnutrition in children under the age of five.^3^ A study from Ethiopia has revealed significant association of maternal education with wasting of children. Furthermore in the same study even father’s education has been documented strongly associated with wasting of children, however in our study father’s education was not found strongly associated with nutritional status of children.^16^ A cross sectional study done in Karachi also has validated significant association of maternal education with nutritional status of children.^4^ Although a substantial number of studies do verify our results regarding importance of maternal education, we came across a study which demonstrated that impact of maternal education is also dependent on other socio-environmental factors.^17^ Contrary to our hypothesis that employment status of mothers can hamper the growth rate of their children, we did not find significant association with poor nutritional status showing maternal employment does not have marked negative impact on growth. It is proved in a study that maternal employment impacts on cognition level also but this affect is trivial and was not universally observed.^18^ Positive effect of maternal employment on growth and development of their children has been documented in various other studies validating our results.^19^,^20^ Promising effect of maternal employment on child’s well-being and nutritional status has also been documented by Hoffman.^21^ Similarly Sattar has documented providential effect of maternal employment on child’s nutritional status.^22^ Endorsement of this fact that women’s autonomy is deeply embedded with their educational attainment and employment status which indirectly impacts on growth parameters of her children has been shown by Senerath as well.^23^

Although in our study we could not prove strong association of family size with nutritional status but many studies have documented strong impact of family size and education on growth parameters of their children.^24^ Susan has demonstrated that birth order and family size carries a substantial effect on children’s growth.^25^ A contemporary study done in Bangladesh has supported the strong association of maternal education, working mothers and family size on child’s well-being and growth.^26^ A study done by Desai S has documented that impact of family size is largely dependent on the socio-economic condition of family.^27^

## CONCLUSION

Maternal education has definite and significant effect on nutritional status of children. This is the key factor to be addressed for prevention or improvement of childhood malnutrition. For this it is imperative to launch sustainable programs at national and regional level to uplift women educational status to combat this ever increasing burden of malnutrition.

### Authors’ Contribution

**AI:** Conceived, designed, statistical analysis and manuscript writing.

**AB:** Designed, reviewed and final approval of manuscript.

**IB:** Data collection and editing of manuscript.

**QM:** Data entry, analysis and manuscript writing.
